# Approval Lag for Ablation Catheters: A Comparative Analysis of Regulatory Timelines in the European Union, United States, and Japan

**DOI:** 10.1111/jce.70248

**Published:** 2026-01-09

**Authors:** Gaku Oguri, Katsuhito Fujiu, Norihiko Takeda

**Affiliations:** ^1^ Department of Cardiovascular Medicine The University of Tokyo Tokyo Japan; ^2^ Department of Advanced Cardiology The University of Tokyo Hospital Tokyo Japan

**Keywords:** ablation catheters, cross‐regional comparison pulsed‐field ablation (PFA), cryoablation devices, medical device lag, regulatory approval timelines, regulatory convergence

## Abstract

**Introduction:**

Approval timelines for ablation catheters differ across major markets, potentially delaying clinical access. This study aimed to quantify approval lags for contemporary ablation catheters across the EU, the US, and Japan.

**Methods:**

We retrospectively identified therapeutic ablation catheters with documented approval dates in the European Union, the United States, and Japan using data from PMDA, FDA, and European notified‐body/manufacturer sources. Non‐therapeutic accessories were excluded. Month‐level approval lags were calculated for each regional pair.

**Results:**

Twenty‐six devices met the inclusion criteria. Mean approval lags were 46.2 months for Japan versus EU, 16.7 months for Japan versus US, and 29.5 months for EU versus US. Medians were lower across all comparisons owing to several long‐lag outliers. Legacy cryoablation catheters showed the greatest delays, whereas recent pulsed‐field ablation systems were approved in closer temporal proximity across regions.

**Conclusion:**

Substantial cross‐regional heterogeneity in ablation‐catheter approvals persists, but recent PFA devices demonstrate that near‐synchronous submissions can narrow the gap. Regulatory convergence and coordinated sponsor strategies may further accelerate patient access.

## 1 Introduction

Catheter ablation is a key therapy for atrial fibrillation (AF) and other supraventricular and ventricular arrhythmias, with global procedure volumes continuing to rise. Advances such as irrigated‐tip technology, contact‐force (CF) sensing, cryoballoon ablation, and pulsed‐field ablation (PFA) have improved procedural safety and efficacy, emphasizing the need for timely access to new innovations.

CF sensing has higher success rates and shorter procedure times [1], while PFA represents a novel energy modality with potential for rapid worldwide adoption [2]. However, ablation device approval timelines differ significantly across major regulatory markets. Furthermore, the recent ESC–EHRA Atlas shows marked cross‐country heterogeneity in arrhythmia‐care capacity, reimbursement, and delivery of EP/CIED services within Europe, indicating that access to guideline‐recommended therapies is not uniform even after market approval [3]. This is referred to as “device lag” in Japan, but is more accurately characterized as approval lag in international contexts. Studies suggest that variations in regulatory requirements, evidence standards, and reimbursement pathways contribute to these delays [4], potentially hindering access to better technologies.

In this study, we quantified month‐level approval lags for contemporary ablation catheters (ACs) across three regulatory jurisdictions and examined their implications for regulatory harmonization and global patient access, recognizing that our descriptive analysis does not directly measure clinical utilization.

## 2 Methods

### 2.1 Design and Objective

In this retrospective, descriptive analysis, we evaluated regulatory approval timelines for therapeutic ACs, focusing on differences across the EU, the US, and Japan.

#### 2.1.1 Devices

Devices were identified using Japan's publicly available Pharmaceuticals and Medical Devices Agency (PMDA) Medical Device Approval Database accessed in July 2026. We searched the “ablation‐type cardiovascular catheters” category, which includes therapeutic ACs approved for clinical use in Japan. From this category, we excluded non‐ablative accessories (e.g., connectors, cables, introducers) and other ancillary components to retain only therapeutic ablation catheters. Only devices with approval dates in all three investigated jurisdictions were included. When an approved device was not retrievable within the PMDA category (because of indexing lag/different entry), the approval month and year were verified from the PMDA records and manufacturer communications.

Our inclusion criteria were as follows:

− Therapeutic ACs employing radiofrequency, cryoballoon, PFA, or endoscopic or laser energy sources.
− Clinical indications for AF or other ventricular or supraventricular arrhythmias.

### 2.2 Documented Approval Dates in All Three Regions

Devices with limited clinical use (e.g., niche and limited‐distribution models) were excluded unless deemed historically or clinically relevant. The final dataset included 26 devices.

#### 2.2.1 Data Sources

‐EU (Conformité Européenne marking): Month–year obtained from Notified Body (NB) certificate (via NB site/European Database on Medical Devices [EUDAMED]; if unavailable, extracted from the Declaration of Conformity [Annex IV] or the Summary of Safety and Clinical Performance documents [SSCP] [Class III]). If such documents were not publicly accessible, the month–year of approval was obtained from manufacturer communications or other verified public sources. NB designation/scope were verified using New Approach Notified and Designated Organizations; dates were used as proxies when direct approval dates were unavailable.

− US: Month and year of Food and Drug Administration decision, including pre‐market approval for Class III devices [5].
− Japan: Month and year of marketing approval from the PMDA [6].

For any records without an approval month, the manufacturer's press releases or other public documents were used to determine the date.

### 2.3 Lag Time Calculation

For each device, pairwise approval lags were computed using:‐JP versus EU = JP approval−EU approval‐JP versus US = JP approval−US approval‐EU versus US = US approval−EU approval (with positive values indicating later approval in the US). Lag times are summarized as mean, standard deviation, and median.

## 3 Results

**FIGURE 1:**
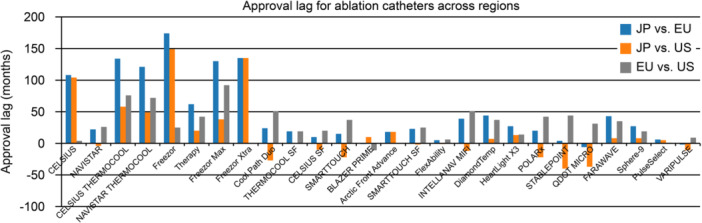
Month‐level regulatory approval lags for 26 ablation catheters across Japan, the United States, and the European Union. Bars show approval lags for Japan versus EU (blue), Japan versus US (orange), and EU versus US (gray); positive values indicate later approval in the first‐listed region. Devices are ordered as in Table 1 based on timing of EU approval.

We screened 47 entries; after excluding accessory or non‐therapeutic items and those not approved across all three regions, and including one device (Sphere‐9™) (Medtronic Inc., Minneapolis, MN, USA) not retrievable via the PMDA category but verified through PMDA approval record, a total of 26 ablation catheters (ACs) remained. The mean approval lags were 46.2 (standard deviation [SD], 52.1) months for Japan versus EU, 16.7 (SD, 47.9) months for Japan versus US, and 29.5 (SD, 25.2) months for EU versus US. The median values were notably lower (23.5 [9.0–73.5], 6.0 [−11.3 to 24.5], and 25.5 [8.3–42.5] months, respectively), reflecting the influence of several extreme outliers, and they should not be interpreted as indicating short approval times because of several lengthy outliers. Several legacy devices (e.g., Freezor™ [Medtronic Inc.]) exhibited delays of > 5 years between the EU and Japan. Conversely, some newer catheters—notably several PFA catheters—tended to show closer temporal alignment in approval across certain regions than historical devices, although substantial variability remained, including marked delays for the FARAWAVE catheter. A graphical comparison of approval lags for all devices is provided in Figure 1 to facilitate device‐level interpretation.

## 4 Discussion

Our analysis highlighted persistent cross‐regional disparities in the timing of AC approvals. Approvals in the EU generally preceded those in the US by approximately 30 months, while approvals in Japan were frequently delayed relative to both. These findings show that approval lags are a global issue influenced by both regulatory processes and manufacturer market‐entry strategies, and, although we did not directly assess clinical availability or patient access, the observed patterns may help identify where regulatory expectations or submission sequencing diverge.

Multiple factors may account for the discrepancies that we identified. Regulatory authorities differ in their expectations concerning the type, quantity, and rigor of clinical evidence required for approval. The US emphasizes randomized data [7], the EU prefers conformity assessments supported by clinical evaluations [8], and Japan often requires supplementary or bridging studies [9] to support approval. Commercial considerations also play an important role. Companies may prioritize submissions to larger or strategically important markets, delaying entry into smaller regions.

Although Japan showed the longest overall delays in this study, variability in approval across devices suggests that regulatory harmonization is achievable in principle. The approval timelines for recent PFA devices were not uniformly synchronized. Rather, some PFA systems were submitted more concurrently across jurisdictions than earlier devices, whereas others exhibited pronounced device‐specific delays, likely reflecting commercial prioritization and strategic considerations rather than inherent regulatory convergence. These patterns imply that convergence is feasible when clinical demand is high and sponsors pursue coordinated submission strategies. Initiatives such as collaborative consultation programs and the International Medical Device Regulators Forum [9, 10] may further facilitate regulatory alignment across regions.

Early radiofrequency catheters required > 10 years for approval in Japan, whereas recent PFA catheters have received authorization within months of being approved in the EU or US. This temporal shift suggests that, although the historical device lag was largely structural in Japan, recent convergence reflects evolving regulatory harmonization and shifting commercial prioritization. At the same time, the persisting variability in approval among individual PFA catheters underscores that synchronized approvals are not yet consistently achieved.

### 4.1 Limitations

In this study, we extracted month‐level approval dates from official regulatory databases and manufacturer sources, and minor ambiguity may remain where agency records did not specify the month. Additionally, approval does not equate to clinical availability, as reimbursement status and adoption patterns were not assessed. The small sample (*n* = 26) and skewed distribution (driven by long‐lag outliers) may affect reliability of summary statistics. Devices not approved in Japan were excluded from analysis, which may have led to an overestimation of approval lag in that region. Moreover, our study required that devices be approved in all three regions; therefore, our dataset may preferentially represent globally prioritized products and, thus, introduce selection bias. The study was underpowered to assess generation‐ or modality‐specific effects, and the small manufacturer‐specific samples limited brand‐level inference. Although the coronavirus disease 2019 pandemic may have influenced regulatory operations during 2020–2023, our data do not allow isolation of pandemic‐related effects, and many devices included were approved outside this period.

## 5 Conclusion

Approval timelines for ACs differ across the EU, the US, and Japan; the EU generally leads, with longer delays observed in the US and Japan. Variability in approval across devices suggests that coordinated submissions may help mitigate approval lag. Accelerating patient access will require both regulatory convergence and sponsor strategies. Future research should extend to other ablation devices and platforms and assess real‐world access (i.e., reimbursement and adoption) across regions. Country‐level analyses may help disentangle regulatory influences from commercial drivers.

**TABLE 1:** Regulatory approval dates and lag times for ablation catheters.

Product name	Manufacturer	EU approval (YYYY‐MM)	US approval (YYYY‐MM)	JP approval (YYYY‐MM)	JP versus EU lag (months)	JP versus US lag (months)	EU versus US lag (months)
CELSIUS® Catheter	Biosense Webster Inc.	1997‐05	1997‐09	2006‐05	108.0	104.0	4.0
NAVISTAR™ Catheter	Biosense Webster Inc.	1998‐04	2000‐06	2000‐02	22.0	−4.0	26.0
CELSIUS® THERMOCOOL® Catheter	Biosense Webster Inc.	1998‐07	2004‐11	2009‐09	134.0	58.0	76.0
NAVISTAR™ THERMOCOOL® Catheter	Biosense Webster Inc.	1998‐11	2004‐11	2008‐12	121.0	49.0	72.0
Freezor™ Cryoablation Catheter	Medtronic	2001‐03	2003‐04	2015‐09	174.0	149.0	25.0
Therapy™ Cardiac Ablation System	Abbott (Irvine Biomedical Inc.)	2002‐05	2005‐11	2007‐07	62.0	20.0	42.0
Freezor™ MAX Cryoablation Catheter	Medtronic	2003‐04	2010‐12	2014‐02	130.0	38.0	92.0
Freezor™ Xtra Cryoablation Catheter	Medtronic	2004‐06	2004‐06	2015‐09	135.0	135.0	0.0
Cool Path™ Duo Ablation Catheter	Abbott	2007‐10	2012‐01	2009‐10	24.0	−27.0	51.0
NAVISTAR™ THERMOCOOL® SF Catheter	Biosense Webster Inc.	2010‐05	2011‐12	2011‐12	19.0	0.0	19.0
CELSIUS® THERMOCOOL® SF Catheter	Biosense Webster Inc.	2010‐05	2012‐01	2011‐03	10.0	−10.0	20.0
THERMOCOOL SMARTTOUCH® Catheter	Biosense Webster Inc.	2011‐01	2014‐02	2012‐04	15.0	−22.0	37.0
BLAZER PRIME™ XP Ablation Catheter	Boston Scientific Corporation	2011‐05	2010‐06	2011‐04	−1.0	10.0	−11.0
Arctic Front Advance™ Cryoablation Catheter	Medtronic	2012‐08	2012‐08	2014‐02	18.0	18.0	0.0
THERMOCOOL SMARTTOUCH® SF Catheter	Biosense Webster Inc.	2014‐07	2016‐08	2016‐06	23.0	−2.0	25.0
FlexAbility™ Ablation Catheter, Sensor Enabled™	Abbott	2014‐07	2015‐01	2014‐12	5.0	−1.0	6.0
INTELLANAV MIFI™ Open‐Irrigated Ablation Catheter	Boston Scientific Corporation	2016‐01	2020‐04	2019‐04	39.0	−12.0	51.0
DiamondTemp™ Ablation System	Medtronic	2017‐12	2021‐01	2021‐08	44.0	7.0	37.0
HeartLight® X3 Endoscopic Ablation System	CardioFocus Inc.	2019‐03	2020‐05	2021‐06	27.0	13.0	14.0
POLARx™ Cryoablation Balloon Catheter	Boston Scientific Corporation	2020‐02	2023‐08	2021‐10	20.0	−22.0	42.0
INTELLANAV STABLEPOINT™ Ablation Catheter	Boston Scientific Corporation	2020‐06	2024‐02	2020‐10	4.0	−40.0	44.0
QDOT MICRO™ Catheter	Biosense Webster Inc.	2020‐08	2023‐03	2020‐02	−6.0	−37.0	31.0
FARAWAVE™ Pulsed Field Ablation Catheter	Boston Scientific Corporation	2021‐02	2024‐01	2024‐09	43.0	8.0	35.0
Sphere‐9™ Mapping & Ablation Catheter	Medtronic	2023‐03	2024‐10	2025‐06	27.0	8.0	19.0
PulseSelect™ Pulsed Field Ablation System	Medtronic	2023‐11	2023‐12	2024‐05	6.0	5.0	1.0
VARIPULSE™ Pulsed Field Ablation Catheter	Biosense Webster Inc.	2024‐02	2024‐11	2023‐12	−2.0	−11.0	9.0

*Note:* Data are presented as YYYY‐MM (i.e., month‐level precision). Lag definitions: JP versus EU = JP − EU; JP versus US = JP − US; EU versus US = US − EU (positive values indicate later approval in the US vs. EU). Sphere‐9™ Catheter: Received approval from the Pharmaceuticals and Medical Devices Agency (PMDA), but domestic clinical introduction in Japan remained limited as of the study's cutoff date.

## Funding

The authors received no specific funding for this work.

## Ethics Statement

The authors have nothing to report.

## Consent

The authors have nothing to report.

## Conflicts of Interest

The authors declare no conflicts of interest.

## Data Availability

The datasets were derived from sources in the public domain. U.S. Food and Drug Administration. Premarket Approval (PMA) database: https://www.accessdata.fda.gov/scripts/cdrh/cfdocs/cfPMA/pma.cfm. Pharmaceuticals and Medical Devices Agency (PMDA). Regulations and Approval/Certification of Medical Devices: https://www.pmda.go.jp/english/review-services/reviews/0004.html.
